# Tension and Other Idioms of Distress Among Slum Dwelling Young Men: A Qualitative Study of Depression in Urban Bangladesh

**DOI:** 10.1007/s11013-021-09735-4

**Published:** 2021-07-30

**Authors:** Syed Shabab Wahid, Malabika Sarker, A. S. M. Easir Arafat, Arifur Rahman Apu, Brandon A. Kohrt

**Affiliations:** 1 Department of Global Health, George Washington University, Washington, DC USA; 2 Division of Global Mental Health, George Washington University, Washington, DC USA; 3 BRAC James P. Grant School of Public Health, BRAC University, Dhaka, Bangladesh; 4 Heidelberg Institute of Global Health, Heidelberg University, Heidelberg, Germany

**Keywords:** Idioms of distress, Depression, Slum, Qualitative, Bangladesh

## Abstract

In low- and middle-income countries (LMIC) it is vital to understand acceptable, comprehensive, and culturally appropriate ways of communicating about mental distress. Diagnostic terminology is rarely used, may be stigmatizing, and is subject to misinterpretation. Local terms, such as idioms of distress, can improve mental health literacy and service delivery. Our objective was to examine lived experience and coping connected to distress and depression in an under-researched population: young men from LMIC urban slums. We conducted 60 qualitative interviews with men (ages 18–29) in Bhashantek slum, Bangladesh. Themes were generated using thematic analysis and grounded theory techniques. The heart-mind (mon), mentality (manoshikota), mood (mejaj), head (matha or “brain”), and body (shorir) comprised the self-concept, and were related to sadness, hopelessness, anger, worry, and mental illness. The English word “tension” was the central idiom of distress. “Tension” existed on a continuum, from mild distress or motivational anxiety, to moderate distress including rumination and somatic complaints, to severe psychopathology including anhedonia and suicidality. Respondents connected “tension” to burnout experiences and mental illness which was summarized in an ethnopsychological model. These findings can inform culturally sensitive measurement tools and interventions that are acceptable to the community, potentially increasing engagement and enhancing therapeutic outcomes.

## Introduction

Depression is a major public health problem across the world affecting an estimated 300 million people globally (Patel et al. 2016). It represents the second leading cause of disability, with an estimated global point prevalence of 4.7 percent, and is the eleventh largest contributor to the global burden of disease (Ferrari et al. 2013). Although more than 80 percent of the people with mental illness reside in low- and middle-income countries (LMIC) (Rathod et al. 2017), the diagnostic categories of depression and its screening and measurement tools, have been developed in high-resource, Western cultural contexts. Research mostly conducted in North America and Europe inform the diagnostic criteria for depression in the Diagnostic and Statistical Manual (DSM) and the International Classification of Disease (ICD), the two prominent psychiatric systems of nosology (American Psychiatric Association 2013; World Health Organization 1992). Standardized measurement instruments, however, can miss out on key aspects of distress as manifested in local contexts (Mendenhall, Yarris and Kohrt 2016). While some core symptoms of depression are common across the world (Haroz et al. 2017), there are key variations attributable to cultural influences, that have clinical and public health implications. Additionally, most psychological treatments are developed in relation to Western concepts and descriptions of depression, and evidence for effectiveness is determined by changes in Western biomedical symptoms of depression (Cuijpers et al. 2008). This raises questions about whether the most important aspects of suffering are being identified and whether interventions are impacting those aspects of life and experience most valued in local settings (Chevance et al. 2020).

As efforts grow to address the global burden of depression, with particular attention to non-Western cultural groups and LMIC, there is a need to understand how mental illnesses are experienced and the cultural constructs that are used to describe such conditions (Kaiser and Jo Weaver 2019). These local experiences and understandings will inform how to better design and implement public health and clinical initiatives that will be effective within specific cultural contexts (Lewis-Fernández and Kirmayer 2019). Exploring and understanding local experiences can account for cultural variability of the signs and symptoms of depression and inform the development of culturally sensitive measurement tools as well (Haroz et al. 2017).

Culture shapes beliefs about the causes people attribute to the manifestation of depression, the subjective experience of the condition, how and which symptoms are communicated, behaviors taken in response, attitudes and decisions about treatment, and subjective evaluation of the efficacy of therapeutic outcomes (Kleinman 1980). These domains often lie outside biomedical frameworks for lay populations in LMIC, and frequently involve the use of culturally and linguistically salient ways of communicating suffering. Haroz and colleagues (2017) recommend “a broader, bottom-up, open-ended approach to better understand the applicability of the DSM depression diagnostic criteria and whether there are other symptoms/features that represent the common experience of depression in populations worldwide.” They discuss the importance of open-ended qualitative research, where “depression is not the starting point” to better understand the condition as it manifests in local contexts. One such approach is to explore and incorporate local ways of how distress is understood and expressed, which was coined by Nichter (1981) as “idioms of distress.”

Idioms of distress are the “socially and culturally resonant means of experiencing and expressing distress in local worlds,” and are often polysemic and dynamic in nature, with continuous transformation due to historic and cultural processes (Nichter 2010). These idioms of distress can be used to communicate internal states across a range of severity, from mild distress to clinical psychiatric morbidity (Lewis-Fernández and Kirmayer 2019). Idioms of distress are often correlated with psychiatric disorders, but also capture information and heterogeneity beyond standard DSM/ICD diagnostic categories (Kohrt et al. 2014). These associations often do not manifest as a one-to-one relationship with psychiatric conditions as outlined in the DSM/ICD, and often exhibit symptoms of more than one disorder (Hinton and Lewis-Fernández 2010).

Hinton and Lewis-Fernández (2010) discuss several ways such idioms of distress hold clinical and public health utility. For depression measurement, the use of idioms of distress in a given culture may indicate the markers of psychopathology in that context. Idioms of distress can also indicate missing aspects of phenomenology, can refine or expand psychiatric nosology, and detect the emergence of new forms of distress (Lewis-Fernández and Kirmayer 2019). A growing body of evidence has demonstrated the successful incorporation of idioms of distress into mental health assessment tools for greater sensitization of psychiatric screening (Cork, Kaiser and White^2019^; Fabian et al. 2018; Ice and Yogo 2005; Kaiser et al. 2013; Weaver and Kaiser 2014).

For psychological treatments, idioms of distress can “identify intervention goals, negotiate treatments, promote engagement, and agree on desired outcomes for recovery” (Lewis-Fernández and Kirmayer 2019). Idioms of distress can be indicative of past exposure to trauma, the risk of future destructive behavior, and the degree of life distress and psychosocial functioning. All of these can aid clinicians’ understanding of patients’ causes of distress and act as the focus of therapeutic interventions, thereby increasing empathy and treatment adherence (Hinton and Lewis-Fernández 2010). A meta-analysis of evidence-based psychotherapies for depression indicated that culturally adapted treatments had better acceptability, and provided some preliminary evidence that interventions adapted to the local context, often using local idioms of distress, were more effective than those that were not (Chowdhary et al. 2014).

Idioms of distress are understood in relation to ethnopsychology, which is the anthropological study of concepts of the self, the mind and the body, and their processes and interrelationships (White 1992). Divorced from broader cultural models of ethnopsychology, idioms of distress can be denuded of their meaning and significance. Ethnopsychology is needed to assure that such terms are understood in context, to identify how these overlap and differentiate, and to successfully incorporate idioms of distress into public health messaging and treatments (Kohrt et al. 2012). As idioms of distress often present with a strong focus on somatization, it is also useful to develop ethnophysiological models, described as ‘‘the culturally-guided apperception of the mind/body rather than actual biological differences’’ (Hinton and Hinton 2002). Combined with ethnopsychology, the exploration of ethnophysiology and the perceived associations with psychiatric disorders can construct more holistic narratives of idioms of distress, and better inform culturally sensitive care provision.

Accordingly, in this study, we explore the lived experience, idioms of distress and ethnopsychology combined with ethnophysiology, of distress and depression, in a population of young men living in an urban slum of Bangladesh, a low resource country in South Asia. We also examine the coping strategies adopted by this population in response to distress. A systematic exploration of the idioms of distress in urban Bangladesh has not yet been conducted to date. Not only is this a contribution to mental health research in South Asia, but the focus on young men also fills an important gender gap since the majority of psychological intervention studies in LMIC have focused on women (Singla et al. 2017). Therefore, documenting men’s idioms of distress and ethnopsychology can inform more gender-equitable mental health services. Finally, the focus on later adolescence and young adulthood is important because, 90 percent of the world’s young people live in LMIC (Nagata et al. 2018), and because depression incidence peaks during this period and can continue as a burden throughout life if left unaddressed (Thapar et al. 2012). Some recent evidence has also indicated that adult mental illness categories may not fully apply to the experiences and symptoms most salient among young people (Midgley et al. 2015; Nagata et al. 2018). Mental health in this age group is now being prioritized by multiple funding bodies as well (Grand Challenges Canada 2020; Wellcome Trust 2020). Considering these multiple factors, it is crucial to understand distress and depression from the perspective of young male populations in non-Western settings to adequately inform culturally salient measurement tools and to improve preventive and curative public health strategies.

## Methods

### Study Setting and Research Team

This study was conducted in *Bhashantek* slum of Dhaka city, Bangladesh. Urbanization, climate change, and persistent poverty in Bangladesh are causing mass migration to cities, leading to burgeoning slum communities (Afsana and Wahid 2013). Slums of Dhaka city are some of the most densely populated places on earth (> 220,000 people per kilometer squared) (Angeles et al. 2009). Slums like *Bhashantek* are rife with risk factors for depression including social exclusion, absence of basic services like sanitation or trash removal, insecure land tenure, legal disempowerment, rampant and persistent poverty, crime and drugs, flooding and fire hazards, and elevated rates and risks of injury, and infectious and chronic disease (Afsana and Wahid 2013; WHO 2010).

There are some linguistic considerations in studying idioms of distress in Bangladesh. The country is largely a monolingual society where the majority of people speak the native language, Bangla (or Bengali). However, due to its historic legacy of British colonialism and spread of the English language through globalization, English has diffused substantially in spoken discourse. In particular, code-switching and code-mixing, which entail the insertion of English words or phrases into Bangla sentences or vice versa, are widely normed aspects of spoken communication across all social strata of Bangladesh (Hasan 2015).

The study team included a doctoral candidate (SSW) in global public health, who is an experienced global mental health qualitative researcher, and two anthropologists (ASMEA, ARA) with considerable qualitative research experience in *Bhashantek*. Other members were a professor of public health (MS) at  BRAC University with decades of research experience in Bangladesh, and a professor of global mental health (BAK) at George Washington University, who is a psychiatrist and a medical anthropologist.

### Ethics

Ethical approval for this study was provided by the institutional review board of the BRAC James P. Grant School of Public Health, BRAC University, Bangladesh.

### Target Population and Sampling

The target population for this study was young men (ages 18–29) living in *Bhashantek* slum of Dhaka city, Bangladesh. Existing quantitative data previously collected (Rabbani et al. 2018) in *Bhashantek* among the target population was used to purposively select respondents using a stratified sampling strategy (Patton 2014). For our sample, we wanted to capture diverse perspectives on how distress is experienced in the study population. Accordingly, two criteria were used to recruit individuals across a range of distress severity: (1) their score on the General Health Questionnaire-12 (GHQ-12), a screening tool comprised of stress and depressive symptomology (Goldberg and Williams 1988); and (2) their membership in three sub-groups based on distress severity, as derived by latent class analysis (LCA). The GHQ-12 is a Likert style questionnaire with scores ranging from 0 to 36, with higher scores indicating poorer psychological well-being. In Bangladesh, a score higher than 11 may indicate the presence of psychological morbidity (Department of Educational and Counselling Psychology 2016). Latent class analysis is a statistical method of classifying individuals from a population into similar groups based on their responses to a set of questions (Garrett and Zeger 2000), like a depression screening questionnaire. The LCA was derived from the GHQ-12 items, and conducted as part of a different study, the results of which have been published elsewhere (Wahid et al. 2021). The three derived latent classes were “Wellness,” “Distressed,” and “Severely distressed.” Accordingly, sampling across these classes allowed us to recruit individuals who were empirically grouped according to severity of symptoms. Please refer to Table 1 for a breakdown of the sample. We oversampled for the “Severely distressed” class to address symptom heterogeneity and increase the likelihood of recruiting potentially disordered individuals.

**Table 1:** Sampling stratified by latent class and GHQ-12 Score

Latent class	Population and sample	GHQ-12: 0–9	GHQ-12: 10–15	GHQ-12: > 15	Total
Severely distressed	*Available population*	*16*	*128*	*67*	*211*
	**Qualitative sample**	**1**	**11**	**14**	**26**
Distressed	*Available population*	*109*	*220*	*0*	*329*
	**Qualitative sample**	**12**	**11**	a	**23**
Wellness	*Available population*	*281*	*3*	*0*	*284*
	**Qualitative sample**	**11**	b	a	**11**
	*Total available population*	*406*	*351*	*67*	*824*
	**Total qualitative sample**	**24**	**22**	**14**	**60**

^a^No individuals met the criteria in the population

^b^Individuals were unavailable or did not consent to interview

### Data Collection

A semi-structured interview guide was developed using the categories from Kleinman’s explanatory model framework of mental illness (Kleinman 1980). These included the causes attributed to distress, lived experience and phenomenology, course and severity, effects on respondents’ lives, and beliefs and behaviors around treatment and coping. We adopted an iterative approach to data collection. First, six pilot interviews were conducted to test and refine the interview protocol. The interview started by exploring the daily routine and background of respondents. Afterwards, we initiated inquiry on distress by eliciting the emotional and mental state of the respondent and any recent stressful life events. Using these events and experiences as anchors we explored the explanatory model categories outlined above. We used common Bangla terms for sadness and worry to probe for underlying distress. After the pilot phase was concluded, we reviewed the raw audio to identify respondent-driven idioms of distress. This analysis revealed ubiquitous use of the English word ‘*tension*’ as the most common idiom of distress. We adjusted the interview protocol by adding specific questions on the nature of ‘*tension’* and its connection to mental illness. 60 qualitative interviews were conducted on-site in 2018–2019 by two of the authors (ASMEA, ARA), both male anthropologists, who had intimate familiarity with the site and long-standing relationships with the community. Interviews were conducted at respondents’ homes or private community spaces in *Bhashantek* that were suggested by participants. Interviews were conducted in the local language and audio recorded with informed consent.

### Data Analysis

Interview audio was professionally translated and transcribed into English for analysis. We generated an *a priori* codebook using relevant theoretical literature. Subsequently, during an open coding process, inductive codes were introduced, until no new codes could be identified. This finalized codebook was used to code the entire dataset. The first author, who is a native Bangla speaker, analyzed the data using NVivo, Version 12 (QSR International 1999), in consultation with the other authors at each stage of analysis.

During and after coding, we applied thematic analysis techniques (Ryan and Bernard 2003) and continually reflected on the data relating codes to one another and contrasting findings to analytic frameworks and social science theories. During this process, ‘*tension*’ was identified as the central focus of the narratives, around which the rest of the data could be organized. Accordingly, we adapted the grounded theory framework proposed by Strauss and Corbin (1990) and situated ‘*tension*’ as the core phenomenon, using axial coding. We then used selective coding and organized the data into the other components of Strauss and Corbin’s framework. These included: (1) Causal conditions – factors which cause the core phenomenon; (2) Strategies – actions taken in response to the core phenomenon. (3) Context and intervening conditions – The broad and specific situational factors that hold influence over causal conditions, core phenomenon, and strategies; and (4) Consequences – which represent the outcomes from adopted strategies.

For the ethnopsychology and ethnophysiology domains, we maintained a database of local words during coding that referred to concepts of the mind and body, and related idioms of distress. After coding was concluded, we conducted lexical searches of these constructs within the data, to ascertain the salience of use by respondents. We used a similar reflective process establishing connections between idioms of distress to inform an ethnopsychological model of ‘*tension’* and mental illness. All words and phrases that were used to denote psychological complaints and idioms of distress were preserved in the translated transcripts in the original Bangla language for analysis.

## Results

### Respondent Demographics

We present respondent demographics including age, schooling, and socioeconomic indicators in Table 2. The majority (52%) of our sample were between 18 and 21 years of age and from the poorest socioeconomic quintile. Most had basic education (57%). About 23 percent of the sample had a GHQ-12 score of 15 or above, and 44 percent were from the severely distressed latent class.

**Table 2:** Demographic characteristics of interview respondents (*n* = 60)

	*N*	%
Education		
No formal education	3	5
Up to 5th grade	23	38
Higher than 5th grade	34	57
Age		
18–21 years	31	52
22–25 years	15	25
26–29 years	14	23
Socioeconomic quintiles		
Poorest	31	52
Poorer	11	18
Middle	7	11
Richer	3	5
Richest	9	14
GHQ-12 Score		
0–9	24	40
10–15	22	37
>15	14	23
Latent class		
Wellness	11	18
Distressed	23	38
Severely distressed	26	44

### Concepts of the Self and Related Idioms of Distress

Respondents frequently used both English and Bangla words and phrases to describe concepts of the self, and related idioms of distress. The aspects of the self included the ‘*mon*’ (heart-mind), ‘*mejaj*’ (temperament), ‘*manoshikota*’ (mentality), ‘*matha’* (literal translation: head; figurative translation: a construct that symbolizes integration of the mental aspects of the mind within its biological physical dimension), ‘*brain*’ (an English word which was used by respondents as analogous to the ‘*matha*,’ as defined above), and ‘*shorir*’ (physical body). It must be cautioned that these terms are not to be interpreted as neatly divided categories. Rather, there was substantial overlap in their conceptual and linguistic meanings and use. ‘*Mon’* was often used in combination with ‘*mejaj*,’ as ‘*mon-mejaj*,’ to reflect the seamless overlap of mind and mood, or with ‘*manoshikotha*,’ as ‘*mon-manoshikota*,’ which illustrates the congealed nature of the emotions and the intellect. We discuss each of these below and provide an overview of the idioms of distress related to these constructs in Table 3.

1. **‘*Mon*’** (heart-mind): ‘*Mon*’ was used by respondents most prominently to denote aspects of the heart and the mind. We combine and operationalize it as the ‘heart-mind.’ ‘*Mon*’ is a concept historically rooted in the ancient Sanskrit and Pali language word ‘*manas*’ and can be found in use today in multiple South Asian countries (Kohrt and Harper 2008)*.* The ‘*mon*’ was referred to by respondents as the seat of emotion.
  “When I feel upset (‘*mon kharap thakle*’), I get dizzy spells sometimes.” – 19-year-old man (GHQ-12 score: 6; Latent class category: Wellness)
2. **‘*Mejaj*’** (temperament or mood): Respondents frequently used ‘*mejaj’* by itself to denote anger, and in conjunction with ‘*mon’* as ‘*mon-mejaj’* to indicate mood or temperament of the heart-mind. ‘*Mejaj*’ was used often with adjectives of temperature (e.g., heat) to describe its state.
  “I was thinking that I couldn’t do anything [financially] in life. Suddenly I became super angry (‘*mejaj ta gorom hoye gelo’*). There was a bamboo next to the bed and I punched it.” – 21-year-old man (GHQ-12 score: 13; Latent class category: Severely distressed)
3. **‘*Manoshikota*’** (mentality): This word was most commonly combined in its adjective form ‘*manoshik*,’ with concepts such as ‘*chaap*’ (pressure) to indicate mental pressure or strain. Respondents often referred to *‘manoshik shomossha*’ (mental problems) and ‘*manoshik rog*’ (mental illness). These terms are used commonly in Bangladesh to refer to mental disorders. Respondents also used ‘*manoshik obosta,*’ which refers to one’s mental state.
  “When I am under mental pressure (‘*manoshik chaap’*) I go to the tea shop and smoke 2-3 cigarettes.” – 20-year-old man (GHQ-12 score: 24; Latent class category: Severely distressed)
4. **‘*Matha*’** (head) and **‘*Brain*’**: Respondents used ‘*matha*’ and the English word ‘*brain*’ to communicate the integrated physical and mental aspect of the mind. We group these together due to the similarity in how these concepts were combined with other words to create idioms of distress that referred to a state of burnout or cognitive overload (Please refer to Table 3). The ‘*matha*’ was also referred to be hot (‘*gorom*’) or cool (‘*thanda*’) to denote anger or even-temperedness, respectively. The ‘*brain*’ was referred to when denoting severe effects of mental illness (e.g., ‘brain stroke’).
  “When I am upset (‘*mon kharap’*) or have ‘*tension*’… if at that time anyone speaks down to me, my head gets heated up (‘*matha gorom hoiya jay’*) … I feel like attacking them. I lose control of my head (‘*matha thik thake na’*)” – 25-year-old man (GHQ-12 score: 8; Latent class category: Distressed)
5. **‘*Shorir****’* (physical body): Respondents most commonly spoke of ‘*durbolata*’ (weakness) of the ‘*shorir*’ or ‘*khoti*’ (bodily harm) as corporeal idioms of distress. The degeneration of the ‘*shorir’* was often described as an effect of mental illness.
  “If someone is facing dangers (‘*khoti*’) to their mental well-being (‘*manoshik obosta*’) or is troubled by too much thinking or is ‘*weak*’ in the mind (‘*mon*’), I don't think they can be considered physically healthy. I don't think their body (‘*shorir*’) will be fine either. Basically, it is through mental strength that the body stays healthy.” – 19-year-old man (GHQ-12 score: 17; Latent class category: Severely distressed)

**Table 3:** Concepts of the self and related idioms of distress in urban Bangladesh

Concepts of the self	Related idioms of distress	Literal translation	Lay interpretation
***Mon*:** heart-mind **Other definitions:** mind; heart; Intellect; understanding; perception; spirit; mood; mental state; mental spirit;	*Mon kharap*	Heart-mind is sad	Sadness
	*Mon bhalo na*	Heart-mind is not good	Sadness
	*Mone chaap*	Pressure in the heart-mind	Stressed; burdened
	*Mon halka*	Heart-mind is light	Unburdened; not stressed
	*Mon bhalo*	Heart-mind is good	Feeling good; not sad
***Mejaj*:** Temperament or mood **Other definitions:** mood (of mind); temperament; temper	*Mejaj kharap*	Mood is bad	Anger; irritability; sadness
	*Mejaj groom*	Mood is heated	Anger; irritable; reactive;
	*Mejaj bhalo na*	Mood is not good	Anger; sadness
	*Mejaj Thanda*	Mood is cool	Calm; even-tempered
	*Mejaj bhalo*	Mood is good	Feeling good; even-tempered
***Manoshikota*:** Mentality	*Manoshik chaap*	Mental pressure	Mental pressure
	*Manoshik shomossha*	Mental problems	Mental problems
	*Manoshik rog*	Mental illness	Mental illness
***Matha*** **Literal**: Head **Figurative**: Representing the integrated physical and mental aspects of the mind **Other definitions:** head; skull; brain; intellect	*Matha noshto*	Head is broken or rotten	A state of burnout or temporary cognitive overload where decision-making, ability to think, or logical reasoning is affected due to psychological stress and being over-burdened
	*Matha kaj kore na*	Head does not work	
	*Matha thik nai*	Head is not okay	
	*Matha aulaiya gese*	Head is jumbled	
	*Matha elo melo*	Head is disorganized	
	*Matha ulta palta*	Head is messy	
	*Matha “hang”*	Head freezes like a malfunctioning software	
	*Mathay chaap*	Pressure in the head	Stressed or over-burdened
	*Matha groom*	Head is hot	Temper; Anger
	*Mathar tar chira*	Wires in the head are torn	Someone who is angry Someone who is unpredictable Someone who is crazy or mentally ill
	*Matha kharap*	Head is bad or spoiled	Crazy or mentally ill
**“*Brain*”** **Figurative**: Used as a synonym of the *matha* (head)	*Brain stroke*	Brain has a stroke	Used to denote an event that causes severe harm to the brain
	*Brain “stop”*	Brain stops working	A state of burnout or temporary cognitive overload where decision-making, ability to think, or logical reasoning is affected due to psychological stress and being over-burdened
	*Brain “hang”*	Brain freezes like a malfunctioning software	
	*Brain kaj kore na*	Brain does not work	
	*Brain noshto*	Brain is broken or rotten	
	*Brain “out”*	Brain stops working	
	*Brain “short”*	Brain has a short-circuit malfunction	
	*Brain er khoti*	Brain gets harmed	Damage to the brain
***Shorir*** Physical body; corporeality;	*Shorir durbol; Shorirer durbolota;*	Weakness in or of the body	Bodily weakness
	*Sharirik shomossha*	Physical problems	Physical problems
	*Shorirer khoti*	Harm to the body	Bodily harm or injury

Definitions provided from the Bengali-English dictionary of the Bangla Academy (Bangla Academy 2015)

### Prominent Domains of Distress

In addition to the idioms of distress that were directly linked to concepts of the self, respondents also discussed a range of other terms. We organized these into six domains: (1) Sadness and low mood; (2) Hopelessness; (3) Irritability and anger; (4) Worry; (5) Severe mental illness; and (6) ‘*tension*.’ There was considerable overlap between these categories as well, indicating the interconnectedness of how respondents experienced distress. We discuss each of these domains below. Definitions from a Bengali-English dictionary are provided as well (Bangla Academy 2015).

1. **Sadness and low mood**: ‘*Dukkho*’ and ‘*Koshto*’ were two commonly used idioms of distress for sadness and low mood. These are closely related conceptually and are defined as (*dukkho*; pg. 306) “sorrow; uneasiness; trouble; difficulty; grief; distress; unhappiness; misery; suffering woe; tribulation” and (*koshto*; pg. 115) “bodily suffering; pain; to suffer; to be distressed; troubled; fatigued,” respectively. Respondents often referred to these together as ‘*dukkho-koshto.*’ These terms were used either independently or situated within the heart-mind (‘*mone koshto*’). Other frequently used idioms of distress for sadness were related to feelings (‘*bhalo lage na*:’ I don’t feel good; or ‘*kharap lage*:’ I feel sad or bad).
  “When my girlfriend’s father wanted to marry her off I felt really sad (‘*kharap lagto*’)…I suffered (‘*koshto*’)…couldn’t share with anyone…” – 28-year-old man (GHQ-12 score: 17; Latent class category: Severely distressed)
  “Even when I’m sad (‘*dukkho*’) I don’t let people know I’m upset (‘*mon kharap*’).” – 19-year-old man (GHQ-12 score: 1; Latent class category: Wellness)

2. **Hopelessness**: The word ‘*hotasha*’ (defined [pg. 857] as disappointed; disillusioned; despondent; despair; hopelessness) was a prominent idiom of distress and was frequently evoked to communicate frustration, disappointment, and hopelessness by respondents. A related word was ‘*bertho*’ (failure; to fail) which was used in reference to internalizing blame and viewing oneself as a failure.
  “The religious holiday is coming up…I have no work, no cash in hand. I have a wife at home, mother, father…I have to give gifts to them, but I can’t afford to… so there is some despair (‘*hotasha*’) regarding this… some ‘*tension*’ also...” – 18-year-old man (GHQ-12 score: 9; Latent class category: Distressed)
3. **Irritability and anger**: Irritability and anger was often reported with an internal affective aspect, or an externalizing aspect, constituting both verbal and behavioral acts. Respondents reported feeling and demonstrating ‘*raag*’ (anger) as a reaction to stressful experiences. The ‘*mejaj*’ (temperament/mood) was most closely related to the irritability domain.
  “I am not feeling too well (‘*mon kharap*’) …I am unemployed…don’t feel good about it…Sometimes I get angry and heated (‘*mejaj gorom*’).” – 22-year-old man (GHQ-12 score: 15; Latent class category: Severely distressed)
4. **Worry**: The worry domain was highly endorsed by respondents. Terms for worry included ‘*chinta*’ or ‘*chinta-bhabna*’ (thoughts; thinking; to worry) or ‘*onek beshi chinta’* (thinking or worrying too much) to indicate repetitive negative thought patterns, ruminative tendencies, and intrusive thoughts. Worry was mostly said to manifest in in the head (e.g., ‘*mathay chinta ashe*:’ thoughts manifest in my head*)*. Respondents also used ‘*chaap*’ (pressure) independently to convey feelings of stress and strain.
  “…Because of ‘*tension*’*.*..not always, but sometimes, when my mind (‘*mon*’) is very weak (‘*durbol*’) when I am thinking too much (‘*beshi chinta*’) then I feel like a failure (‘*bertho*’).” – 18-year-old man (GHQ-12 score: 11; Latent class category: Severely distressed)
5. **Severe mental illness**: Respondents used several idioms of distress such as ‘*pagol*’ (mad), ‘*matha kharap’* (literal translation: head is bad; figurative translation: crazy), ‘*tar chira’* or ‘*mathar tar chira’* (literal translation: wires in the head are ripped), and an English word ‘*mental*’ to describe a mental state or person with significant impairment and externalizing symptoms of acute mental disorder. These ‘*pagol*’ or ‘*mental*’ individuals were described by respondents as speaking gibberish, sleeping in the gutter, not maintaining hygiene, forgoing clothing, forgoing eating or sleeping, or eating and sleeping at unnatural times, etc. Severe mental illness was most closely associated with malfunctions of the ‘*matha*’ (head) and the ‘*brain*.’
  “I know this one guy whose younger brother went almost insane (‘*pagol*’) with ‘*tension*’ and pressure (‘*chaap*’). When we ask him something, he answers after a very long pause. Apparently, he had an accident, and he was doing fine with medicine but after he got married, he became ‘*mental*.’” – 26-year-old man (GHQ-12 score: 0; Latent class category: Wellness)
6. **‘*Tension*’**: This English word was the most salient and frequently discussed idiom of distress by respondents, and used most commonly as an idiom of worry:
  “‘*Tension*’ means to think (‘*bhabna*’) about certain things. We have to worry (‘*chinta*’) about different things in our head (‘*matha*’). Any worry on a particular situation is what ‘*tension*’ is. Worrying about something in particular is called ‘*tension*.’” – 19-year-old man (GHQ-12 score: 13; Latent class category: Severely distressed)

*‘Tension*’ was also the most central idiom of distress, connected to almost all other forms of distress. It was described either as a cause or an effect for any of the other terms in Table 3 (e.g., ‘*tension*’ could lead to sadness, or pressure could lead to ‘*tension*’). Other terms were described to combine to form ‘*tension*’ (e.g., worries and pressure) or ‘*tension*’ in combination with other terms could lead to the manifestation of another idiom of distress (‘*tension*’ and pressure could lead to ‘*brain hang*;’ or too much ‘*tension*’ could cause one to turn into a ‘*mental*’). Therefore, we classify ‘*tension*’ as its own category due its considerable multivocality that encompassed a wide range of meanings. Accordingly, to address the key role of ‘*tension*’ in Bangladeshi ethnopsychology, we examine the etiology and lived experience of ‘*tension*’ and its connection to mental illness in detail in the following sections.

### ‘Tension’

**Fig. 1:**
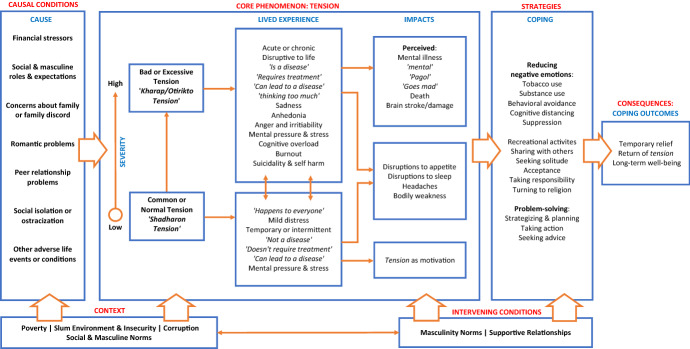
‘Tension:’ A grounded theory adaptation

We organize this section on ‘*tension*’ according to the grounded theory adaptation presented in Fig. 1. We discuss the major themes including the causes of ‘*tension*,’ lived experience and ethnopsychology of ‘*tension*,’ and treatment and coping for ‘*tension*.’ We frame these domains in the context and intervening conditions, as appropriate.

### ‘Tension:’ Causal Conditions

Respondents described a wide array of sociocultural and economic causes that lead to ‘*tension*.’ Financial stressors were the most strongly endorsed cause. Respondents described living in conditions of rampant poverty, working in low paying insecure jobs, operating vulnerable street businesses, and often having crippling financial debts. Some described experiencing police raids which would cause business shutdowns or loss of stock. Similarly, others described frustrations with bribes being required in acquiring jobs or having jobs filled by those with connections to local power structures. Financial stressors were amplified for respondents as they were older adolescents or young adults transitioning from a relatively responsibility-free youth, to having family expectations around contributing to daily household costs, and taking care of older parents, younger siblings, and their wives and children. These expectations were heavily influenced by social and masculine norms which dictate men, especially the older male child, to assume the mantle of the provider, and caused significant distress in respondents if this expectation could not be met. Accordingly, financial problems often seeded conflicts within families. One respondent shared:

“I had to drop out of school because my financial condition was very bad. There was a lot of financial pressure. The state of my mind (‘*mon*’) hasn’t been very good for the past couple of months. I knew that I wouldn’t have this job for long. For that I felt really bad. I’m still feeling bad (‘*kharap ekhono lagtese*’). I don’t know whether I will get another job or not – I feel ‘*tension*’ about that. I am married now, and people gossip [regarding not being able to fulfill responsibilities]. This happens if one is unemployed. That’s why I am feeling uneasy…[probing]…I don’t know if I will be able to afford food in a few days, I keep thinking about this…I don’t know brother...I am in ‘*tension*’ because of this.” – 21-year-old man (GHQ-12 score: 13; Latent class category: Severely distressed)

Another major source of ‘*tension*’ was romantic problems. Many respondents described on-going strife in a relationship or the experience of a break-up, as causing considerable ‘*tension*.’

“The ‘*tension*’ was firstly because I had struggle to come visit her and maintain the relationship. Secondly, I used to worry (‘*chinta*’) whether our relationship will survive when my family meets her. After the relationship ended, I had ‘*tension*’ thinking ‘Why did this happen even after I struggled so hard to make it work?’ … After she broke up with me, I tried to kill myself.” – 24-year-old man (GHQ-12 score: 16; Latent class category: Severely distressed)

Even though most respondents reported having friends, many narrated feelings of isolation or loneliness, because they could not open up to friends to share emotions or problems, which was cited as a source of ‘*tension.*’ This was heavily framed by masculine norms of not wanting to face ridicule if perceived as weak or emotional. Problems and fights among friends or being socially ostracized were also cited as causing significant distress. Some respondents directly lamented the life circumstances that had forced them and their families to live in slums. Finally, some respondents cited experiencing adverse life events as a cause of ‘*tension*.’ These included destruction of homes or businesses due to fires or slum demolition drives by municipal authorities, death or debilitating disease of a family member, and personal illness or disability. One respondent shared:

“When I have worries (‘*chinta-bhabna*’) I get so much ‘*tension*’ that I can’t sleep. I stay awake the whole night. I keep thinking (‘*chinta*’) if I could be as happy as other people are with their families. I think of many other things. I wish that I could be like any other normal person. I stammer when I talk. People make fun of me…my heart breaks! So, I think it’s better not to talk. My friends also laugh at me and tease me. I tell them not to make fun of my weakness, but they keep on teasing me. My eyes fill up with tears…I was born like this! I also think about my family. We live in a slum – it is government land. If they evict us, I’m not sure where we would go. When I am alone my ‘*tension*’ comes. I think I have let my family down [by not earning]. My younger brother [also worries me] – he’s sick, weak in health. I don’t know if I can assist [financially] in his treatment.” – 18-year-old man (GHQ-12 score: 13; Latent class category: Severely distressed)

### ‘Tension:’ Lived Experience

In order to capture the range of distress described as ‘*tension*’ we differentiate between what respondents described as ‘*shadharon tension*’ or common or normal ‘*tension*,’ and ‘*kharap/otirikto tension*’ or bad or excessive ‘*tension*.’ One respondent described this distinction:

“Well, if a person has really bad, deep ‘*tension*’ on an issue, and goes into *'depression'*- to get out of that ‘*tension*’ he does many things like drinking, drugs, physically harming himself…and goes to a level that he becomes totally insane. That’s mental illness to me. The kind of ‘*tension*’ I have is like normal, not excessive. I don't think it's an illness. It hasn’t gone beyond control.” – 18-year-old man (GHQ-12 score: 6; Latent class category: Wellness)

Respondents reported that one could have both kinds of ‘*tension*,’ simultaneously due to different reasons, or that common or normal ‘*tension*’ could eventually (but not necessarily) turn into bad ‘*tension*,’ over time, if corrective measures were not taken. These were not described as distinct or static categories and are best interpreted as lying on a continuum of distress.

### Common or Normal *‘Tension’ (‘Shadharon Tension’)*

Respondents who were sampled for having less distress mostly endorsed this type of ‘*tension*.’ They stated common or normal ‘*tension*’ as causing mild distress, something that “is normal” or that “happens to everyone,” and which consisted of every day worries and difficulties. In the distress spectrum, at its mildest, the nervousness experienced before a competitive soccer match, or the prospect of having to stay awake at night to perform ritual prayer, were examples described as causing this form of ‘*tension*.’ Most commonly reported sources were general concerns about career, family, studies, etc. One respondent shared:

“If her [girlfriend’s] phone is switched off I get angry. Because it’s off…I can’t reach her, so I get ‘*tension.*’ I start to think if I did anything wrong that caused her to switch off the phone.” – 20-year-old man (GHQ-12 score: 4; Latent class category: Wellness)

The effects of this type of ‘*tension*’ were reported to be mostly somatic in nature, with minor disruptions to sleep, appetite, and bodily pains, most frequently cited. Thinking too much and rumination were referenced commonly as well:

“When these thoughts (‘*chinta*’) come, I twist and turn in bed. I cannot sleep. I stay awake till 1 AM sometimes, due to ‘*tension*.’” – 19-year-old man (GHQ-12 score: 1; Latent class category: Wellness)

Respondents said that they could easily overcome this type of normal ‘*tension*,’ and that it was temporary or intermittent in nature. Normal ‘*tension*’ was closely associated with non-pathological worries. Accordingly, these respondents did not feel any treatment was necessary for ‘*tension*.’ One interlocutor shared:

“I don’t think ‘*tension*’ or worry lead to any diseases so there really is no need for doctors or treatments. I personally was in some ‘*tension*’ - but the more you think of these things, the more these thoughts come. Best to just focus on work and move on.” – 26-year-old man (GHQ-12 score: 0; Latent class category: Wellness)

Some respondents actually shared that this type of normal ‘*tension*’ was necessary to motivate one to pursue goals and achieve things in life. However, respondents cautioned about excessive ‘*tension*.’

“The ‘*tension*’ that I feel is for my own good. In one sense ‘*tension*’ harms people. In another sense, if people aren’t in ‘*tension,*’ they cannot achieve anything in life. One cannot be successful, because if I don’t worry (‘*chinta*’) about the future or have ‘*tension*’ about the future, I won’t be able to do anything in the future. I think ‘*tension*’ is good for some people and bad for some people. Too much ‘*tension*’ (‘*otirikto tension*’*)* is not good. It is not good to have ‘*tension*’ all the time. – 24-year-old man (GHQ score: 16; Latent class category: Severely distressed)

### Bad or Excessive *Tension *(*Kharap/Otirikto Tension*)

In contrast to normal ‘*tension*,’ excessive or bad ‘*tension*’ was endorsed mostly by respondents sampled for having higher severity of distress. They described ‘*tension*’ as a severe and distressful condition with a host of cognitive, affective, and somatic experiences. This form of ‘*tension*’ was often referred to as being acute and chronic. Most respondents reported persistent low mood, feelings of sadness, and hopelessness. These affective states were most commonly reported to be related to the heart-mind (‘*mon*’) and mentality (‘*manoshikota*’):

“Yes, my heart-mind is sad (‘*moner koshto*’). There is some trouble with my family. There are worries (‘*chinta*’). I mean after coming back home after working all day and seeing my family suffering…when I see their pain (‘*koshto*’) I don't feel very good (‘*bhalo lage na*’), I feel really bad (‘*khub kharap lage*’) ... I feel restless…I feel bad and also get ‘*tension.*’ Like I was saying, my mental condition (‘*manoshik obosta*’), I stay depressed most of the time… (‘*bishonno thaki, maximum time*’) – it’s there because of my financial situation. I can’t support myself and my family – I stay in ‘*tension.*’ This is the primary state of my mind (‘*moner obosta*’) these days. I feel quite hopeless (‘*hotasha*’) – 26-year-old man (GHQ-12 score: 18; Latent class category: Severely distressed)

Anhedonia was reported by most respondents with this form of bad/excessive ‘*tension*,’ and some also reported feelings of worthlessness and disruptions to functionality. Respondents reporting severe symptoms spoke of negative impacts on almost all aspects of the self, including the heart-mind (‘*mon*’), head (‘*matha*’) and body (‘*shorir*’). One respondent described the anhedonic state of his mind and impact on his functionality:

“If only I knew why I feel like this. Nothing specific comes to mind, no answer for my restlessness. When I feel restless the thought that comes in my head is: ‘I don’t have anything!’ I feel like I want to die. I don’t feel good anymore. My head stops functioning (‘*matha kaj kore na*’), and I don’t feel like working. I have to struggle hard to keep my spirits up and under control until work ends. Because of my mental condition (‘*manoshik obosta*’) I didn’t work for 4 months, and just stayed home. My wife was so angry. She eventually had to take a job. Entire days would pass, and I wouldn’t go to work. Just sat in the house hibernating, not interacting with anyone. It feels like my body (‘*shorir*’) has lost its strength.” – 25-year-old man (GHQ-12 score: 9; Latent class category: Distressed)

Many respondents mentioned that they often had intrusive thoughts or fantasies of wanting their life to end, which they attributed to bleak life circumstances. However, most of these individuals stated that they did not plan to actually attempt suicide. At the most extreme end of the spectrum, suicidal attempts or self-harming were reported by few highly distressed individuals:

“When my wife left, it was a ‘*tension*.’ After she left me, I drank poison. First, I took rat poison, then I took some pills. It was powerful brother…after 5 minutes, I could feel it burning inside. I felt like vomiting. Then my friends came and took me to the hospital.” – 24-year-old man (GHQ-12 score: 16; Latent class category: Severely distressed)

Respondents also reported significant irritability and anger arising because of ‘*tension*’ or co-arising with ‘*tension*.’ The aspect of self that respondents related to irritability was the ‘*mejaj*’ (temperament/mood). Many men described experiencing substantial anger due to stressful events and conditions and engaging in verbal fights with family, with female members often being recipients of that anger. Some respondents also reported engaging in both verbal and physical altercations with other men or displays of physical anger against inanimate objects due to ‘tension’ (e.g., punching the wall). When asked to describe how he felt when he had ‘*tension*,’ one respondent said:

“My behavior changes when I am in ‘*tension*.’ Sometimes I get angry and scream (‘*raaga-raagi*’) at my younger sister. Now I might misbehave but later perhaps I'm crying [because of that] when I am alone.” – 24-year-old-man (GHQ-12 score: 16; Latent class category: Severely distressed)

Somatic complaints of appetite loss and insomnia were universally reported symptoms, but were described to be more severe, disruptive, and “a problem” for this type of ‘*tension.*’ The concepts of self closely tied to somatic aspects of bad/excessive ‘*tension’* were the ‘*shorir*’ (body) and the ‘*matha*’ (head). One respondent shared:

“Physical problems (‘*shorir er shomossha*’) happen due to ‘*tension*.’ I cannot sleep. I also have disrupted sleep, waking up frequently at night. I don’t have any desire to eat. I have appetite and do eat a little, but due to ‘*tension*’ and lack of sleep it feels different.” – 24-year-old-man (GHQ-12 score: 16; Latent class category: Severely distressed)

Disruptions to sleep were strongly connected to worrying or thinking too much. Excessive rumination and somatic complaints were described as the most common features of bad/excessive ‘*tension*.’ Other cognitive effects such as loss of concentration were cited by a minority of respondents as well. ‘*Tension*’ in the form of rumination was said to come mostly at night, or when respondents were alone. Rumination was often connected to things that were causing low mood and sadness. One man described his ‘*tension*:’

“Some thoughts (‘*chinta*’) will come and keep spinning around in my mind (‘*mon*’). These thoughts keep on winding and twisting in knots. Whatever is making me sad (‘*mon kharap*’), that keeps on spinning in my head (‘*matha*’).” – 25-year-old man (GHQ-12 score: 10; Latent class category: Severely distressed)

This type of bad/excessive ‘*tension*’ was perceived by respondents as being significantly dangerous and causing harm to the body and the mind. One respondent described these impacts:

“…from ‘*tension*,’ many people can have stroke…some people die slowly due to ‘*tension*’*…*some lose hope and fall into despair (‘*hotasha*’) and commit suicide… ‘*tension*’ causes many deaths…” – 18-year-old man (GHQ-12 score: 16; Latent class category: Severely distressed)

When queried if bad or excessive ‘*tension*’ required treatment, respondents were split in their perceptions on whether treatment was necessary. Some believed that there was no treatment for ‘*tension*’ and recommended talking to friends and self-management, while others mentioned traditional healers, or medicine for insomnia and headaches. Many respondents connected psychiatric care to ‘*tension*.’ One man mentioned:

“Yes, it needs to be treated. In the doctor’s office, I see many people seeking treatment to reduce ‘*tension*.’ They take a medication named Benzit [local antidepressant brand]. I have seen my friend taking medication for ‘*tension*.’ He takes a Benzit and falls asleep, all relaxed. It’s not a sleeping pill but it opens up the cells of the ‘brain’ (‘*brain er cell guli khuila dey*’). One can go to psychology doctor (‘*psychology daktar*’) as well.” – 18-year-old man (GHQ-12 score: 6; Latent class category: Wellness)

### *‘Tension*’ and the Connection to Mental Illness: An Ethnopsychological Model

**Fig. 2:**
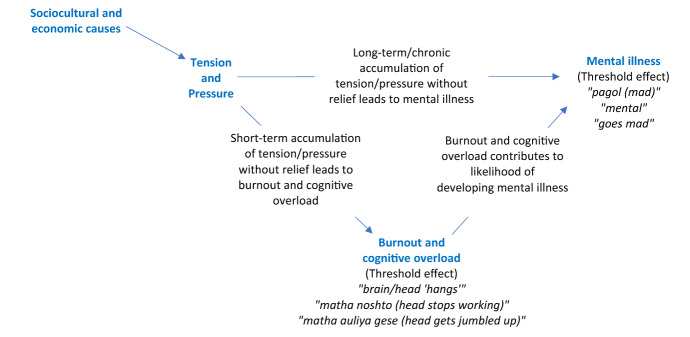
An ethnopsychology of ‘tension’ and mental illness

In this section we propose an ethnopsychological model of 'tension' and mental illness (Fig. 2). We specifically asked respondents to comment on the connection between ‘*tension*’ and mental illness. Some respondents endorsed ‘*tension*’ as a mental illness in itself. Most explained that ‘*tension*’ led to mental illness. Respondents cited socioeconomic causes described previously as creating ‘*tension*’ and mental pressure (‘*chaap*’) to accumulate over time. In the short-term, having excessive *tension* and pressure were said to lead to a temporary state of burnout or cognitive overload that respondents described using a variety of vivid idioms (Table 3) of the ‘*mon*,’ ‘*matha*,’ and the ‘*brain*.’

“…worrisome thoughts used to come to my mind when my father was ill…I couldn’t eat or sleep. I was so worried, my head felt broken (‘*amar matha noshto*’).” – 20-year-old man (GHQ-12 score: 11; Latent class category: Severely distressed)

“… due to ‘*tension*’…due to pressure (‘*chaap*’) one’s head gets jumbled up (‘*matha aulaiya jai*’).” – 19-year-old man (GHQ-12 score: 10; Latent class category: Distressed)

These states were described to temporarily impact decision-making, disrupt the ability to think clearly, or clearly assess or address problems. Eventually, excessive ‘*tension*’ or burnout could lead to mental illness, respondents stated:

“If one has too much ‘*tension*’… it may lead to mental illness (‘*manoshik rog*’). ‘*Tension*’ has a limit, for everyone. Say, I can lift 1 Kg but if you give me 10 Kg, I will be under pressure (‘*chaap*’). For this reason, my brain can get broken *(*‘*brain noshto*’) …because of the ‘*brain*’ being broken, mental illness can happen…Because of too much pressure (‘*otirikto chaap*’)” – 29-year-old man (GHQ-12 score: 16; Latent class category: Severely distressed)

Left untreated, the consequences of excessive ‘*tension*’ or pressure were said to lead to the states of ‘*pagol*’ *or ‘mental*,’ the most impaired psychopathological states associated with mental illness by participants:

“Yes, it can happen [‘*tension*’ leading to mental illness] …because of worries (‘*chinta-bhabna*’) there can be intense pressure (‘*chaap*’). If one can’t cope with it, he may go crazy (‘*pagol*’) …because of too many worries (‘*chinta*’), one becomes mad (‘*pagol*’*)*.” – 23-year-old man (GHQ-12 score: 15; Latent class category: Severely distressed)

“I have heard that from ‘*tension*’ one can have many problems…one gets dark lines under the eyes, doesn’t eat, and sleeps less. Due to ‘*tension*’ there’s pressure (‘*chaap*’) on the ‘*brain*’ and one can become ‘*mental*.’” – 19-year-old man (GHQ-12 score: 10; Latent class category: Distressed)

We summarize and present these connections between *tension*, pressure (*chaap*), states of burnout and cognitive overload, and mental illness in an ethnopsychological model in Fig. 2.

#### Coping in Response to ‘*Tension*’

Respondents adopted a host of strategies to cope with both normal and excessive/bad ‘*tension*.’ We organized these strategies into two categories: (1) reduction of negative affect, and (2) problem-solving strategies. Respondents described stressors or ‘*tension*’ causing substantial unpleasant feelings to arise. In response, they reported adopting a multitude of coping strategies to reduce such unpleasant emotions and return to a state of equilibrium. Some stressors, interlocutors said, could not be resolved immediately and needed to be systematically addressed. For this latter type of stressor, they reported adopting problem-solving strategies. While a wider variety of emotion-focused coping was reported, both problem-solving approaches and negative affect reduction strategies were reported evenly by respondents in terms of frequency. Respondents often reported taking on both types of coping simultaneously (e.g., studying as a problem-solving strategy to prepare for an exam, while smoking cigarettes as an immediate strategy to reduce related fears and worry).

For reducing negative emotions, smoking was by far the most commonly endorsed strategy. Some respondents also indicated engaging in substance use to deal with ‘*tension*.’

“Nothing really makes me feel better. Sometimes perhaps getting high or drinking makes me feel a little better. It makes me feel different, helps to feel at ease and pass the time. Cigarettes make me feel less ‘*tension*.’” – 18-year-old man (GHQ-12 score: 14; Latent class category: Distressed)

Adaptive emotion-focused strategies that provided immediate relief included engaging in recreational activities such as playing sports, watching movies, or browsing the internet. Others referred to God as looking out for them in times of difficulty. Some maladaptive cognitive strategies such as suppressing feelings, cognitively distancing from stressors, and avoidance were commonly reported. For others, a process of accepting the stressor (e.g., the end of a romantic relationship), positive reappraisal of a situation, and taking psychological responsibility were strategies that were tied to better outcomes in the long-term.

“Sometimes I think if I were born in an affluent family, I could afford many things…but I don’t allow myself to think anything negative. My mom says, ‘Whatever God has given us is good. I am trying to provide for you within my means.’ This really had an effect inside me – that whatever little we have is okay.” – 18-year-old man (GHQ-12 score: 6; Latent class category: Wellness)

Many respondents reported seeking solitude and silence away from sources of ‘*tension*’ as a preferred coping strategy.

“When there are problems at home, I don’t feel like staying there. I go find some place quiet and sit by myself. I just sit at the field all by myself and watch children play.” – 19-year-old man (GHQ-12 score: 6; Latent class category: Wellness)

Discussing one’s problems with others was heavily influenced by norms of masculine strength and infallibility. Some participants reported an unwillingness to disclose their troubles due to the likelihood of losing respect or facing ridicule from others.

“I don't like to share. I keep everything inside. You see, sharing can get you into trouble…For me, I like to analyze, ask questions in my mind, search for answers, etc. That is much better. If I share with someone, then they may take it negatively, or gossip with others.” – Respondent-38 (GHQ-12 score: 17; Latent class category: Severely distressed)

For others, however, sharing troubles with close family members and friends was an acceptable and effective way to cope. This demonstrates that adherence to masculine norms and the availability of supportive relationships acted as moderators in determining if an individual felt comfortable in opening up and discussing their problems with others. Accordingly, these two factors were added under “Intervening Conditions” in Fig. 1. Intervening conditions in this population determined if certain coping strategies were adopted (or not).

“I didn't let that ‘*tension*’ get to me. I stayed busy with work all day. The ‘*tension*’ didn't bother me. And I have like 4 or 5 close friends. I talked to them. I shared and discussed my problems. All of these things contributed to my ‘*tension*’ going down.” – 26-year-old man (GHQ-12 score: 18; Latent class category: Severely distressed)

Problem solving strategies reported by respondents included strategizing and planning or engaging with friends and family to develop solutions to issues that were causing ‘*tension*,’ or directly taking action to address the stressor.

“My ‘*tension*’ is only money related. I think about it for 10-20 minutes – how to solve it? That’s what I try to do. Who can I approach for assistance? I try to plan with him.” – 28-year-old man (GHQ-12 score: 9; Latent class category: Distressed)

Most coping strategies that focused on reducing negative feelings had immediate effects on reducing ‘*tension.*’ However, in most cases for such strategies, ‘*tension*’ returned over time, making it a temporary outlet for feeling good. It was only through the resolution of a stressor (e.g., repaying a loan; accepting the end of a romantic relationship) that respondents reported they experienced well-being in the long-term.

“Sometimes if I do something good, get a good result of my actions, then I feel good. Suppose we get an order in the business, and we successfully supply the materials – I feel good. I can support the family, my mom feels happy, and I feel good.” – 26-year-old man (GHQ-12 score: 17; Latent class category: Severely distressed)

## Discussion

This qualitative study among young men in an urban slum of Bangladesh revealed a range of terminology and experiences related to mental health. These include lay notions of the aspects and processes of the body and the mind, and how disruptions to these can affect well-being. The terms capture an interconnected network of cognitions, emotions, behaviors, and outcomes related to wellness and suffering. In particular, the findings reveal the central role of ‘*tension*,’ a polysemic, non-stigmatized local idiom of distress, that was ubiquitously used to communicate a range of distress, and its perceived role in the pathway to developing severe mental illness.

The findings on ‘*tension*’ have important clinical and assessment implications. The clinical word for depression in Bangla (‘*bishonotta*’) was raised only by one respondent in our interviews, while ‘*tension*’ was universally described. ‘*Tension*’ at its severe end was reported to consist of signs and symptoms that matched DSM diagnostic criteria for major depressive disorder, including *anhedonia, low mood, suicidality, irritability, appetite problems*, and *worthlessness or guilt*. Similar to Haroz and colleagues’ (2017) findings on the experience of depression around the world, we see an almost complete absence of *psychomotor agitation or retardation* descriptions, and low presence of descriptions related to *concentration problems*, *fatigue,* or *impaired function* in this population. Respondents also placed strong focus on somatization (i.e., appetite and sleep disruptions), which aligns with specific findings for South Asian populations. As reported by Kaiser et al. (2015), we find universal endorsement of ‘thinking too much’ as an aspect causing significant distress. Additionally, similar to global populations (Haroz et al. 2017), we find high endorsement of the presence of anger and irritability, which is perhaps moderated by male gender in our findings.

Our findings on the ethnopsychology of ‘*tension*’ and its perceived connection to mental illness align with what others have observed on the time course and either trait-like or continuous nature of idioms of distress (Lasater et al. 2018). Hinton and Lewis-Fernandez (2010) discuss that some idioms of distress can be “near-continual,” while others are episodic, with continual constructs often preceding others, along a continuum of severity. They demonstrate this with the example of ‘*nervios*’ a Caribbean-Latino idiom of distress. Individuals could suffer from ‘*nervios*’ or have “altered nerves” which over time, or ‘chronic *nervios*,’ could lead to ‘*locura*’ (madness), indicating a continual progression. Individuals could also suffer from an attack of nerves (‘*ataque de nervios*’), an episodic condition. There are parallels to how respondents in the current study described the accumulation of ‘*tension*’ and pressure to lead to either an episodic burnout state of cognitive overload, or as a continual process, to severe mental illness over time. In an ethnopsycholoy study of idioms of distress in Kenya (Mendenhall et al. 2019), respondents described a continuum starting with ‘thinking too much,’ progressing onto stress, burnout, leading to depression, and eventually on to madness. Interestingly, this progression seemingly aligns with the findings summarized in our ethnopsychological model of ‘*tension*’ and pressure, leading to burnout/cognitive overload and severe mental illness, and could be pointing to similar underlying processes across these vastly different contexts. Cross-cultural comparisons of ethnopsychology studies examining distress and depression can be an interesting direction for future research.

The findings of this study strongly indicate social and masculine norms in Bangladesh to be critical drivers of distress, disorder, and increased vulnerability to developing psychiatric morbidity for young men. Bangladeshi society remains structured according to traditional patriarchal ideals of hegemonic masculinity (Islam and Karim 2012). Previous research with both men and women on the masculine role in Bangladesh found endorsement of characteristics such as assertiveness, dominance, courage, daring, aggressiveness, sexual prowess, control over one’s wife, and the ability to maintain strict discipline over one’s family, as masculine ideals (Hollerbach, Khan and Khan 2014; Khan and Townsend 2014). In particular, there are strong social expectations for the man to be the primary breadwinner and serve as the head of the household (Islam and Karim 2012; Khan and Townsend 2014). Under the conditions of chronic poverty, men often find it difficult to live up to these standards (Selim 2010), and financial strain has been found to be a strong predictor of depression among men in Bangladesh (James-Hawkins et al. 2019). This can explain the attribution of financial stressors as the dominant cause of ‘*tension*’ by respondents in the current study. Adherence to masculine norms of the provider likely exacerbates the considerable financial strain already inflicted by conditions of poverty and deprivation for this population.

Norms surrounding masculine dominance is another key aspect of interest in Bangladesh and has been associated with depression among men as well (James-Hawkins et al. 2019). Masculine dominance is characterized by the use of aggression to maintain one’s reputation, exhibiting sexual prowess, and maintaining control over one’s wife or other female family members (Levant 2011). The respondents in the current study reported experiencing significant distress and ‘*tension*’ at the deterioration or end of a relationship with a female partner. Upholding norms of masculine dominance likely aggravates the distress from a breakup or divorce, as it is also a public display of ineffective ‘control’ over a woman, a normative violation with reputational consequences for young men. Although we probed considerably on the sexual prowess domain, respondents did not endorse this to be a source of ‘*tension*’ or distress.

In our results, however, we find that not all young men endorse rigid ideals of masculine dominance. Some young men report strong relationships with close friends and family members, including mothers, sisters, and wives. Such relationships were characterized by trust and open communication regarding problems and vulnerabilities. In a social network analysis conducted with this population (a different component of this research project with young men in *Bhashantek* slum), Rabbani et al. (2018) find that social connection indeed plays a role in providing a buffer against mental distress through psychosocial support from one’s peers, adding a degree of validation to the qualitative findings of the current study. This is in contrast to some respondents who reported a reflexive rejection of open communication with others, in fears of reputational damage and being perceived as “weak,” which is aligned with norms of masculine dominance.

It may also be useful to analyze the findings of this study through the lens of gender strain theory, which has three components specific to masculinity (Levant and Powell 2017). We find substantial evidence of *discrepancy strain* which posits that men experience distress if they fail to live up to their internalized ideals regarding manhood. As discussed above, struggles to meet the gender-defining role of the provider and head of the family in Bangladesh was the most critical source of distress reported by respondents. We also find some degree of *dysfunction strain* which states that endorsement of harmful social norms will lead to negative consequences for men in a given culture. Dysfunction strain has been studied previously regarding substance use by men (Liang et al. 2017). In our findings, we find masculine norms surrounding unfettered use of cigarettes to deal with ‘*tension*’ as being universally endorsed, which has substantial negative implications for health outcomes for men in the long term. Finally, we can posit that there are elements of *trauma strain* which is experienced by men belonging to a marginalized class. We find that inhabiting slum environments, chronic poverty, deprivation, and absence of opportunities for upward mobility substantially marginalize these young men, as reported in their narratives of socioeconomic frustrations.

The findings of this study can inform the development of culturally salient assessment tools for ‘*tension*.’ Due to the considerable heterogeneity of meaning that individuals assigned to ‘*tension*’ we discourage an approach that seeks to build a standardized scale of ‘*tension*’ in Bangladesh. Instead, a client-centered psychometric instrument like the Psychological Outcome Profiles (PSYCHLOPS) may be better suited (Ashworth et al. 2004). The PSYCHLOPS is valuable as it allows for patient-generated definitions of conditions and assessment over time across the domains of problem severity, functionality, course, and subjective well-being. Similar to the PSYCHLOPS, a ‘*tension*’ tool could ask respondents to give their definition of ‘*tension*’ as well as rate the impact of severity in their life. An open-ended response option to define ‘*tension*’ is vital because of the heterogeneity in the interpretation of the construct.

In previously conducted research, scales for ‘*tension*’ have been developed separately among North Indian women (Weaver and Kaiser 2014) and immigrant Bangladeshi women living in the United States (Karasz et al. 2013). Our results indicate that incorporation of men’s experiences need to be explored in such efforts, as ‘*tension*’ may not be a phenomenon restricted to women. Also, the multi-vocality of ‘*tension*’ needs careful examination from the perspective of both distressed and non-distressed populations in contexts where the term is in use. This is necessary to establish the degree of both clinically salient and non-psychopathological experiences that may be subsumed under the term. Given the tremendous heterogeneity of the ‘*tension’* construct with men of a limited age range all located within one community in this study, we caution against generalizing the meaning of ‘*tension*’ within and across populations in one context, or from one context to others.

Indeed, a growing body of literature indicates that the concept of ‘*tension*’ is not a phenomenon bound to any one context. Recent research from across South Asia has reported on ‘*tension*’ as an idiom of distress, including studies from India (Halliburton 2005; Weaver 2017; Weaver and Hadley 2011), Nepal (Clarke et al. 2014; Rai et al. 2017), Bhutanese refugees (Chase Liana, Welton‐Mitchell and Bhattarai 2013), Bangladesh (Selim 2010), and Pakistani immigrants in the United States (Tirodkar et al. 2011). While worry was a central component of ‘*tension*’ in most of these studies, there was notable variation, suggesting that while the term has spread in use across South Asia, there is heterogeneity in its phenomenology, across and within cultures. The current study contributes to this literature by clearly illustrating the heterogeneity of ‘*tension*’ and is an important cautionary note to many of the current global mental health applications that reify idioms of distress into tools or one-size-fits-all interventions.

This emergence of ‘*tension*’ as a ‘regional’ South Asian expression of distress is an interesting addendum to the idea of how ‘global’ idioms of distress (e.g., stress) become ‘localized’ (Mendenhall et al. 2019). Shared cultural aspects across South Asia, driven by common regional mass media and entertainment (e.g., Bollywood), have created the conditions for the spread of culture and ideas, and potentially, common ways of expressing distress. Future research can consider exploring the meanings and implications of ‘*tension*’ across South Asian nations to see if multi-country experiences can be distilled to core elements that are common across the region.

Our findings on the multivocality of ‘*tension*’ have implications for mental health care provision in Bangladesh. We found use of ‘*tension*’ across a continuum of distress within our study population. Interestingly, at its most benign form, ‘*tension*’ was spoken of as a quality that motivates one to achieve goals, similar to how moderate levels of anxiety can be helpful for motivating success in academic, professional, and other areas of life. Although ‘*tension*’ was used to communicate mild to severe forms of distress, often in the same discussion, it was universally recognized and accepted as a non-stigmatized experience that was common for everyone. This has both advantages and disadvantages for clinical and public health considerations. The major advantage is that psychosocial interventions can be adapted to incorporate ‘*tension*’ without stigmatizing those who avail such services, thereby increasing community acceptability. The disadvantages are two-fold: First, there is a risk that clinicians may reflexively substitute it for a clinical condition, or automatically assume the non-pathological nature of ‘*tension*’ and dismiss narratives of ‘*tension*’ as non-clinical distress or mild motivational anxiety. Secondly, public health initiatives offering services for ‘*tension*’ run the risk of pathologizing and stigmatizing the term in communities if it is consistently and exclusively associated with extreme disorder during interventions. Accordingly, for public health implementers, interventions need to be developed, and staff need to be sensitized, to minimize any stigmatizing effects of messaging and community-based activities. For care-providers, narratives of ‘*tension*’ would warrant deeper exploration to probe for any underlying disorder symptoms. During therapeutic encounters or other psychological interventions, ‘*tension*’ can be valuable as an entry point for the discussion. Care providers can then extract the client’s perspective to gain a shared and accurate understanding on what ‘*tension*’ represents for each individual. It is critical to avoid making assumptions either equating or outright substituting ‘*tension*’ for depression or stress, etc. Using the patient’s language by incorporating ‘*tension*’ and other idioms of distress, and drawing from the ethnopsychology of ‘*tension*’ and mental illness as presented in the current study, can increase cultural sensitivity of care provision. The Cultural Formulation Interview, as incorporated in the DSM-5, which can build around the patient’s understanding of ‘*tension*’ could be a useful tool for this purpose (American Psychiatric Association 2013; Lewis-Fernández, Aggarwal and Kirmayer 2020).

Our results do indicate specific tendencies unique to some men, which involve the masculine framing of stoic non-sharing of internal distress in fear of losing respect or being perceived as emotionally weak. For such groups, interpersonal or group therapy interventions may be less acceptable. Alternate approaches of addressing distress and depressive symptoms which involve developing cognitive reassessment and emotional regulation skills through mindfulness interventions, could be potentially well-suited to this population. Such interventions have shown comparable efficacy to cognitive-behavioral therapy (Hofmann and Gómez 2017). As respondents often report seeking solitude and silence to cope with ‘*tension*,’ mindfulness interventions could be a natural fit, and could offer an individual-level solution adhering to masculine norms and coping strategies currently in use by this population. As some men do engage with trusted friends for coping, as confirmed by social network methods by Rabbani and colleagues (2018), community-based interventions using trained peer mentors to deliver depression care (Joo et al. 2016) may be worth examining as well.

However, interventions at the individual level alone may not be sufficient to alleviate distress in such impoverished communities. Initiatives need to be formulated that address the structural and socioeconomic root causes cited by respondents as the sources of distress. In our results, we verify the notion that idioms of distress can raise awareness of social and structural inequities that create and perpetuate the conditions that lead to distress (Kohrt et al. 2004; Lewis-Fernández and Kirmayer 2019). Respondents strongly attributed crippling financial conditions as the major source of their suffering. Social protection mechanisms, microcredit schemes, or cash transfer programs complementing vocational training programs for skill-development could be provided to these populations to break the vicious cycle of poverty and associated mental health problems. In addition, slum environments are a structural representation of poverty and powerlessness and contribute to perpetuating distress. Therefore, adequate infrastructural initiatives need to be developed to offer relocation to secure and dignified housing for urban slum populations. Although such an initiative was launched in 1975, it has unfortunately stalled several times due to systemic challenges within bureaucratic institutions (Mohit 2012).

## Conclusion

In this study we present qualitative findings of idioms of distress in urban Bangladesh from a population of slum-dwelling young men. We present a host of local terms which are used to communicate varying degrees of distress, as connected to local concepts of the self, within the broader sociocultural context. We also present an exposition of the central idiom of distress, ‘*tension*,’ including its etiology, phenomenology, and associated outcomes. We consolidate the results by providing an ethnopsychological model of how ‘*tension*’ is connected to a breadth of human emotions and experiences, including but not limited to mental illness. The results of this study can be used to inform the development of culturally sensitive assessment tools and prevention and treatment programs for the millions of slum-dwelling men in Bangladesh.
